# Molecular characterization and genome sequencing of selected highly resistant clinical isolates of *Pseudomonas aeruginosa* and its association with the clustered regularly interspaced palindromic repeat/Cas system

**DOI:** 10.1016/j.heliyon.2025.e41670

**Published:** 2025-01-06

**Authors:** Hekmat A. Owaid, Mushtak T.S. Al-Ouqaili

**Affiliations:** a Department of Biology, College of Science, University of Anbar, Ramadi, Iraq; b Department of Microbiology, College of Medicine, University of Anbar, Anbar Governorate, Ramadi, Iraq

**Keywords:** CRISPR/Cas, *P. aeruginosa*, Superbug, Carbapenemases, Whole genome sequencing

## Abstract

The presence of the clustered regularly interspaced short palindromic repeats (CRISPR)/Cas system in the superbug *Pseudomonas (P) aeruginosa* presents a unique opportunity to precisely target and edit bacterial genomes to modify their drug resistance. The objective was to detect the prevalence of CRISPR in extensively and pan-drug-resistant *Pseudomonas aeruginosa* and to determine the utility of whole-genome sequencing (WGS) for the analysis of the entire genome for such strains. The antimicrobial susceptibilities of one hundred isolates were assessed using the antibiotic susceptibility test (AST) card of the VITEK system. The presence of the CRISPR/Cas system was determined via specific primers using conventional polymerase chain reaction (PCR). Further, WGS was conducted using a DNA nanoball sequencing platform via BGI-Tech for the isolates of interest. Out of 54 resistant *Pseudomonas aeruginosa* isolates*,* 33 (33.0 %) were metallo-β-lactamase producers. Cas1, Cas3, CRISPR1, and CRISPR2 were positive in 6.0 % of isolates, while incomplete CRISPR1-Cas systems alone were found in 15.0 %. Also, CRISPR2-type was found intact in 26 % of isolates. The prevalence of resistance to antimicrobials in *P. aeruginosa* isolates was significantly greater in the CRISPR/Cas-negative group compared to the CRISPR/Cas-positive. Significant relationships for variables were examined using Fisher's exact tests using Chi-squared and a P-value of <0.05 as a statistical threshold. Further, on examination of CRs as a collective entity, encompassing both extensive drug resistance (XDR) and pan-drug resistance (PDR), it becomes evident that the vast majority of these strains (n = 29; 87.8 %) lacked CRISPR/Cas systems. In phylogenic analysis, PDR-*P. aeruginosa* revealed a very close evolutionary relationship with those originating from Kazakhstan, while XDR was globally unique. Further, the entire genome showed the presence of unique virulence and resistant pseudomonal genes. The CRISPR/Cas system and drug resistance are antagonistic to one another. XDR and PDR *P. aeruginosa* represent a potential threat to public health and contribute to the seriousness of associated illnesses by leading to resistant infections. Further, WGS for the two strains revealed resistance to multiple antibiotics. It is important to examine specific antimicrobial resistance (AMR) pathways, which suggests that a significant number of resistant genes in these isolates indicate a loss of CRISPR genes in the two strains. Furthermore, the WGS approach can lead to a better understanding of the genomic mechanism of pseudomonal resistance to antibiotics.

## 1 Introduction

The rapid evolution of superbugs that are resistant to antibiotics has become one of the most pressing of global public health concerns in recent years [1]. One of these superbugs is the gram-negative bacillary-shaped *P. aeruginosa*, which can be both extensively drug resistant (XDR) and pan-drug resistant (PDR) in nature [2]. A significant proportion of genes resistant to antibiotics identified in *P. aeruginosa* have been transferred from other bacteria, predominantly through self-transferrable plasmids, which are detected as the original sources of antibiotic resistance in numerous bacteria [3]. It is now recognized that a major threat to human health has resulted from the overuse of these antibiotics in healthcare settings since multidrug-resistant bacteria and viruses have proliferated worldwide. To avoid issues linked to multidrug-resistant bacteria (MDRs) without harming beneficial bacteria, a new and unique way of treating such infections has been developed using the CRISPR/Cas genome-editing technique [4].

Production of carbapenemase and extended-spectrum lactamase (ESBL) are two of the main resistance mechanisms that *P. aeruginosa* has developed [5]. Numerous β-lactam antibiotics, such as penicillins, cephalosporins, monobactams, and carbapenems, with global prescribing prevalence, can be inactivated by distinct variants of these enzymes [6]. Also, infections induced by *P. aeruginosa* strains that produce ESBLs are conventionally managed via the administration of carbapenem antibiotics. Nevertheless, the prevalence of carbapenem-resistant *P. aeruginosa* has increased dramatically worldwide, necessitating the substitution of carbapenems with alternative medications like tigecycline and colistin; however, these medications currently represent the sole viable therapeutic choice for managing infections caused by XDR bacteria [7].

Clustered regularly interspaced short palindromic repeats (CRISPR) and their encoding genes, as a defense mechanism, are used by many bacterial species use to ward off foreign genomes, plasmids, and resistance genes [8]. The CRISPR/Cas system consists of Cas genes located upstream, and a collection of foreign DNA sequences referred to as spacers. These spacers originate from bacterial cell invasions by foreign plasmids and phages; their successive addition to the CRISPR array indicates that the bacterial cell has acquired a "memory fragment" of such invaders. Bacteria use the Cas protein's nuclease activity to cleave the DNA sequences that match their spacers [9]. Numerous research efforts conducted over the last five years have shown that antibiotic resistance is inversely related to the presence of the CRISPR/Cas system in the genome of certain bacteria, specifically *P. aeruginosa* [10]. Nevertheless, the findings in this particular domain have occasionally led to conflicting outcomes, hence highlighting the necessity for further investigation in this realm [11].

This study investigates the correlation between the existence of antibiotic resistance factors and CRISPR loci, specifically carbapenemases and ESBLs, in MDR-, XDR-, and PDR-*P. aeruginosa*. It also aims to detect the genetic variation and the existence of the CRISPR/Cas system using whole genome sequencing technology in selected strains.

## 2 Methods

### 2.1 Ethics statement

Written informed consent was obtained from all study participants, which included both patients and their parents, according to the questionnaire laid down by the medical committee of the ethics at the University of Anbar, Iraq, who gave their approval for this study. The university order, with approval number 14 of March 15, 2022, was used and carried out according to the Helsinki Declaration.

### 2.2 Study design and patients

In this cross-sectional study, a total of 175 clinical specimens were collected during the period from February to October 2022, in Ramadi Teaching Hospital, Al-Anbar Governorate, Iraq. Of these specimens, 100 isolates were bacteriologically identified as *Pseudomonas aeruginosa* from the following forms of specimen: ear swabs from otitis media patients (11 %), sputum from patients with repository tract infections (17 %), urine samples from catheterized units (27 %), and wound swabs from burn, osteomyelitis, and diabetic foot infection (45 %) patients.

### 2.3 Bacteriological identification of *P. aeruginosa* isolates

The same hospitals' microbiology labs handled the processing and culturing of the clinical specimens and carried out all bacteriological analyses and confirmatory biochemical testing. Eosin methylene blue agar, MacConkey agar, and Blood agar (Germany, Darms) were used to cultivate the specimens, all of which were incubated for 24–48 h at 37 °C. The typical microbial and biochemical tests for *Pseudomonas aeruginosa*, like lysine iron agar, gram stain triple sugar iron agar, urea/citrate consumption, ornithine decarboxylase, and the oxidase test, were supplemented with morphological and cultural characteristics to allow for isolate identification [12]. *P. aeruginosa* isolates were identified definitively and confirmatively using VITEK®2-g negative identifier (GN ID) cards and the VITEK®2 Compact B System (Marcy-l'Etoile, France, BioMérieux) [13].

### 2.4 Antimicrobial sensitivity test

According to guidelines laid down by the Clinical Laboratory Standard Institute (CLSI), 2024, and the data supply in the VITEK®-2 Compact B System (BioMérieux, France) with VITEK 2 AST-GN cards by Advanced Expert System (AES), appropriate antibiotics for *Pseudomonas aeruginosa* were selected. Additionally, the minimum inhibitory concentrations (MICs) for the selected antimicrobial agents were also determined.

Results are given as minimum inhibitory concentrations using the VITEK®2 Compact B System (BioMérieux, France), whilst the CLSI guidelines were used to decide whether bacteria were susceptible, intermediately susceptible, or resistant [14]. The European Committee on Antimicrobial Susceptibility Testing provides guidelines for interpreting the tigecycline antibiotic susceptibility test (AST) results [15]. To ensure the quality of the antibiotic susceptibility testing, *Escherichia coli* American-type culture collection (ATCC) 25922 was used as an internal quality control.

The study isolates were identified as multidrug resistant when they exhibited resistance to a minimum of three different antibiotic classes, according to their susceptibility profiles. When bacteria were found to be resistant to at least one antibiotic across six different classes, they were referred to as XDR. When all antibiotics tested were unsuccessful in killing the bacteria, the strain was deemed to be PDR. Further, the specific AST card also gives the contributing resistance mechanisms to the antimicrobial agents of choice [16].

### 2.5 Molecular assay

#### 2.5.1 DNA extraction

The SaMag-12TM automated nucleic acid extraction system was used to isolate genomic DNA using a DNA extraction kit (SaMag, Cepheid, Italy). The quantity of the extracted nucleic acid was determined using a Quantus™ Fluorometer (Promega, USA) in order to evaluate the sample quality [17].

Amplification of CRISPR/Cas genes using PCR:

The CRISPR/Cas genes were identified via the polymerase chain reaction (PCR) technique using the particular primers [18] indicated in Table 1.

**Table 1:** The sequences of the primers used in this study.

Gene Primers	DNA sequence (5ʹ→3ʹ)	Target	Product size (bp)	Melting temperature
**Cas 1**	**F-** GCTGTTTGTCAAAGTTACCCGCGAACTC	Cas1 gene	208	66.7
	**R-** GTTTTGATCGCCTCATGAGTCACAGTTG			66.2
**Cas 3**	**F-** GGGTTTCGCTACAAAATCAACATGCCATCG	Cas3 gene	506	67.1
	**R-** CACGAGTTTTTTACGCTCATCAAACCAGAGCG			68.2
**Cas 9**	**F**- acgccaattggttgaaactc	Cas9 gene	225	60.0
	**R**- acgacggcattaagatacgc			60.0
**Cse1**	**F**- CAGTTTAACCGATATTTTCAGCCAGCCGG	Cse1 gene	347	66.36
	**R**- CATCAGTTAATTGCTGCTGTTGCTGACTTTCG			67.02
**I-E-CR 1**	**F**- CTGGCATAACGCCACCGG	incomplete CRISPR1-Cas systems	898–3700	61.2
	**R**- GAGACCCGGTTCTTCGGGC			62.6
**I-E-CR1**	**F-** CAGTTCCTGCAACCTGGCCT	intact CRISPR1-Cas systems	Variable	62.7
	**R-** CTGGCAGCAGGTGATACAGC			61.09
**I-E-CR2**	**F**- GCGCTACGTTCTGGGGATG	For incomplete CRISPR2-Cas systems	522–2850	60.8
	**R**- CGTCGCAAAACTCGACCAGA			60.9
**I-E-CR2**	**F-** GTAGCGAAACCCTGATCAAGCG	intact CRISPR2-Cas systems	Variable	61.8
	**R-** GCGCTACGTTCTGGGGATG			60.8
**I-E-CR3**	**F-** GACGCTGGTGCGATTCTTGAG	intact CRISPR3-Cas systems	Variable	66.0
	**R-** CGCAGTATTCCTCAACCGCCT			66.0

Amplification of Cas1 and Cas3 was achieved using PCR. The conditions followed were initial denaturation for 5 min at 95 °C, 35 cycles of denaturation for 1 min at 94 °C, annealing for 30 s at 60 °C, and extension for 1 min at 72 °C, with a final extension phase of 10 min at 72 °C. For Cas9 and Cse1, gradient PCR was used to titrate the optimal annealing temperature between 54 and 66 °C. PCR primers for all CRISPR-related targets were equally amplified using a common recipe, namely an initial denaturation at 95 °C for 5 min, 35 cycles of denaturation at 94 °C for 1 min, annealing at 63 °C for 1 min, and extension at 72 °C for 1 min, with a final extension at the same temperature for 10 min.

Thereafter, gel electrophoresis, with a 1.5 % agarose gel containing 10 % ethidium bromide, was used to separate the PCR products. The electrophoresis was performed in a 1X Tris/Borate/EDTA (TBE) buffer at 50 V for 5 min and 100 V for 1 h. The predicted size of the PCR amplicon band was confirmed using a 100-base-pair DNA ladder. Fermentas (USA) was employed for comparative analysis of the PCR products' band via Ultraviolet Transilluminator (Vilber Lourmat, Marne-la-Vallée Cedex 3, France). The primers used in the PCR to detect the frequency of CRISPR/Cas system components are reported in Table 1.

#### 2.5.2 Whole genome sequencing (WGS)

WGS was used for the two selected clinical isolates of *P. aeruginosa.* The DNA nanoball sequencing platform from BGI-Tech (Hong Kong) was used. Concentrations were determined via a fluorometer (Qubit Fluorometer, Invitrogen). The purity of the samples and their integrity were checked using agarose gel electrophoresis (concentration of agarose gel: 1 %; voltage: 150 V; electrophoresis time: 40 min). Covaris randomly fragmented 1 μg of genomic DNA. The genomic DNA fragments were carefully selected to have an average size of 200–400 bp using an Agencourt AMPure XP-Medium kit. After being end-repaired, the fragments were 3′ adenylated, and then had adaptors ligated to their ends. The adaptor-containing fragments from the prior stage were amplified in this manner. The PCR products were purified using the Agencourt AMPure XP-Medium kit. The double-stranded PCR products were circularized using the splint oligo sequence after being heat-denatured. Finally, ssCir DNA, or single-strand circular DNA, was employed as the library format; the library passed the quality control test. The libraries that met the appropriate criteria were sequenced using the BGISEQ-500 system. The DNA nanoballs (DNBs) that were created via rolling-cycle replication included more than 300 copies of the reference material. A high-density DNA nanochip was used to load the DNBs into the patterned nanoarray. The final step was to use combinatorial Probe-Anchor Synthesis (cPAS) to get 100 bp reads on both ends.

#### 2.5.3 Bioinformatics **analysis**

Genome assembly and annotation were achieved using the bacterial bioinformatics resource center (https://www.bv-brc.org/), which was also used for the phylogenetic analysis via PATRIC. The representative and reference genomes were provided by PATRIC and incorporated into the phylogenetic analysis included in the Comprehensive Genome Analysis report. To detect a genome's phylogenetic position, the PATRIC global protein families (PGFams) were chosen from the nearest reference and representative genomes found using Mash/MinHash. Nucleotides for each of the protein sequences from these families were mapped to the protein alignment after the sequences were aligned using muscle. After merging the sets of amino acid and nucleotide alignments into a single data matrix, RaxML was employed for analysis. The phylogenetic tree's support values were generated using fast bootstrapping.

The contigs were uploaded to Pathogen Watch (https://pathogen.watch) to confirm species identification and to detect antibiotic-resistant genes. Analysis of virulence genes was achieved using Abricate (https://github.com/tseemann/abricate). The PATRIC Genome Annotation Service employs a k-mer-based approach to detecting AMR genes. This method makes use of the curated collection of representative AMR gene sequence variants maintained by PATRIC. It assigns a functional annotation, the drug class, the broad mechanism of antibiotic resistance and, in some circumstances, the specific antibiotic to which the AMR gene confers resistance to each antibiotic. The genome maps of strains *P. aeruginosa* (PA) −1 and *P. aeruginosa* (PA) −2 were visualized via Proksee [19].

#### 2.5.4 Statistical parameters

The Statistical Package for the Social Sciences (SPSS, IBM Corporation, Armonk, NY, USA) version 20.0 was used for statistical investigation of the data, in particular to determine frequencies, percentages, and means. The significant relationships for variables were examined using Fisher's exact tests and the Chi-squared test (P-value <0.05).

## 3 Results

### 3.1 Susceptibility to antimicrobial agents

**Fig. 1:**
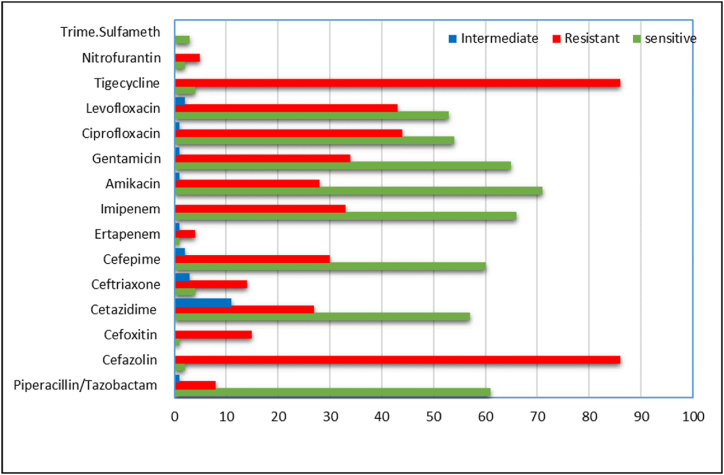
A brief summary of the percentage of *P*. *aeruginosa* isolates that were found to be resistant to antimicrobials based on their MIC values.

A total of 100 *P. aeruginosa* isolates were obtained from a diverse range of clinical specimens. As depicted in Fig. 1, a significant proportion of the isolates exhibited susceptibility to various β-lactam antibiotics, encompassing carbapenems and β-lactam/β-lactamase inhibitors. Moreover, the occurrence of resistance was prevalent with various antimicrobial agents, including fluoroquinolones and aminoglycosides. A significant proportion of the strains exhibited resistance to tigecycline and cefazolin, while just a small percentage of strains (6 % and 4 %, respectively) were found to be sensitive.

The study found that out of 100 isolates, 42 % (42/100) produced extended-spectrum β-lactamase (ESBL), as determined by their responses to various antibiotics and the outcomes of diverse phenotypic assays. All bacteria that produce ESBLs exhibited resistance to multiple drugs, indicating MDR. Moreover, the study revealed that a total of 33 % (33/100) of the isolates exhibited resistance to carbapenems, with all these isolates demonstrating the production of metallo-β-lactamase (MBL) out of the total number of cases (n = 33) examined in the study. Of 100 study isolates, 20 (20 %) were identified as MDR, 22 (22 %) being classified as PDR, while the remaining 11 (11 %) were classified as XDR. Further, 47 (47 %) of the study isolates exhibited sensitivity to nearly all of the study's antimicrobial drugs.

### 3.2 CRISPR/Cas system element distribution of *P. aeruginosa* isolates

CRISPR1, CRISPR2, and CRISPR3 encoding genes were recognized via PCR. The clear existence of CRISPR/Cas was noted in the study isolates. The presence of the type I-E CRISPR1 system in the selected *P. aeruginosa* strains was observed. Primers located in the Cas1 gene were utilized due to this gene being known to be crucial for the CRISPR/Cas system to function.

According to the data presented in Table 2, it can be seen that certain strains possessed either the CRISPR component or the Cas component, while others exhibited both components. Consequently, the presence of the CRISPR/Cas system was identified in 6 % of the isolates. Various gene combinations were identified. Also, it is important to note that 53 % of the bacteria examined displayed either CRISPR or Cas alone, while 47 % of the isolates tested were negative for both.

**Table 2:**
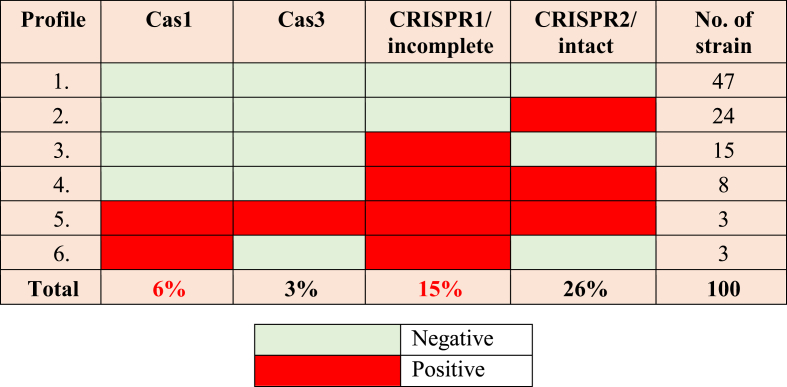
Polymerase chain reaction with an emphasis on detecting Cas and CRISPR genes in *P. aeruginosa* strains, including those carrying either one gene or both, and those lacking CRISPR-encoding genes.

The six strains that were found to be positive for both CRISPR and Cas included a combination of sensitive strains lacking the MDR phenotype (n = 2), CR (n = 4), and MDR-ESBL (n = 4). After amplification of CRISPR/Cas system-encoding genes, they were detected in proportions of 6 %, 3 %, 15 %, and 26 % for Cas1, Cas 3, CRISPR1/incomplete, and CRISPR2/intact, respectively.

### 3.3 The incidence of CRISPR/Cas in nosocomial *P. aeruginosa* isolates and its correlation with antimicrobial resistance

The interference of the CRISPR/Cas system with plasmid transformation and stability, which frequently harbor drug-resistant genes, has been demonstrated. Consequently, we attempted to determine the presence of any potential correlation between the occurrence of CRISPR/Cas in clinical strains and drug resistance profile. The PCR analysis demonstrated that six of the Cas1 PCR-positive strains, accounting for 6 % of the samples, exhibited the presence of at least one CRISPR array, namely CRISPR1 or CRISPR2. The CRISPR/Cas system was found to have a prevalence of 4 % among carbapenem-resistant germs, which concurrently showed resistance to additional antibiotics due to their classification as either XDR or PDR strains. The CRISPR/Cas system exhibited a prevalence of 4 % (n = 4) in the ESBL-producing bacteria but was observed in only 2 % (n = 2) of the non-MDR strains. This observation suggests a negative link between the resistance phenomenon and prevalence of the CRISPR/Cas system.

Since the CRISPR/Cas system is not widely present in *P. aeruginosa*, it is possible that it plays a role in negating the acquisition of drug-resistant genes. The prevalence of this system among the susceptible isolates was found to be 2 % (n = 2) for both CRISPR1/Cas and CRISPR2/Cas. In contrast, the prevalence of CRISPR1/Cas and CRISPR2/Cas in ESBL isolates was found to be 4 % (n = 4) for each of the resistant isolates. Regarding the isolates that produce MBL, 4 % of isolates (n = 4) were found for both CRISPR1/Cas and CRISPR2/Cas.

### 3.4 Relationship between the prevalence of the CRISPR/Cas system and drug resistance

The prevalence of resistance to antimicrobials in *P. aeruginosa* isolates was significantly greater in the CRISPR/Cas-negative group compared to the CRISPR/Cas-positive. The isolates that tested positive for the CRISPR/Cas system exhibited resistance to piperacillin/tazobactam (0 %), cefazolin (4 %), cefoxitin (0 %), ceftazidime (4.2 %), ceftriaxone (0 %), cefepime (4.2 %), imipenem, ertapenem (4.2 %), amikacin (2.8 %), gentamicin (2.8 %), ciprofloxacin/levofloxacin (2.8 %), tigecycline (5.6 %), nitrofurantoin (0 %), and trimethoprim/sulfamethoxazole (0 %). On comparison, the resistance rates to the indicated drugs were found to be higher in the CRISPR/Cas-negative isolates, at 8.3 %, 81.9 %, 12.5 %, 20.8 %, 16.7 %, 23.6 %, 2.8 %, 29.2 %, 22.2 %, 27.8 %, 40.3 %, 38.9 %, 81.9 %, 5.6 %, and 0 %, respectively. CRISPR deficiency is more frequently observed in resistant strains, albeit without statistical significance.

The findings of our study indicate that CRISPR2 is the most prevalent in bacteria that exhibit resistance (n = 19; 19 %), followed by CRISPR1 (n = 8; 8 %), and CRISPR3 (n = 0; 0 %). Conversely, in strains without the MDR phenotype, CRISPR1 was shown to be the most common (n = 7; 7 %), followed by CRISPR2 (n = 7; 7 %), and CRISPR3 (n = 0; 0 %). According to the data reported in Table 3, the most notable disparities were observed for the XDR and MDR groups, since a majority of these strains exhibited a lack of any CRISPR/Cas system.

**Table 3:** The correlation between multidrug-resistant, extensively drug-resistant, and pan-drug-resistant and CRISPR/Cas systems prevalent in isolates of *P*. *aeruginosa*.

MDR/XDR/PDR	CRISPR/Cas Positive No. (%)	CRISPR/Cas Negative No. (%)	Number of strains
**MDR**^**1**^	**0** (**0.0)**	20 (1**00.0)**	**20 (20.0 %)**
**XDR**^**2**^	2 (1**8.2)**	**9** (8**1.8)**	**11 (11.0 %)**
**PDR**^**2**^	**2** (9.**1)**	**20** (90**.9)**	**22 (22.0 %)**
**CR**^**3**^	**4** (1**2.1)**	**29** (87**.9)**	**33 (33 %)**

MDR: Multidrug resistance; XDR: Extensively drug-resistance; PDR: Pan-drug resistance; 1 All MDR isolates produced ESBLs; 2 The XDR and PDR categories mainly comprised metallo-β-lactamase producers, carbapenemases (CRs); while 3 represents the combination of PDR and XDR.

Due to the relatively small sample sizes within each group, it was difficult to make any clear distinction between non-MDR and PDR strains. Consequently, the restricted availability of data hindered the ability to make accurate statistical comparisons. Nevertheless, upon examining CRs as a collective entity, encompassing both XDR and PDR, it becomes evident that the vast majority of these strains (n = 29; 87.9 %) lacked CRISPR/Cas systems. Table 3 reveals the distribution of CRISPR and Cas elements and systems in isolates of *P. aeruginosa*, along with statistical comparisons.

#### 3.4.1 Fundamental statistics of the genome assembly

**Fig. 2:**
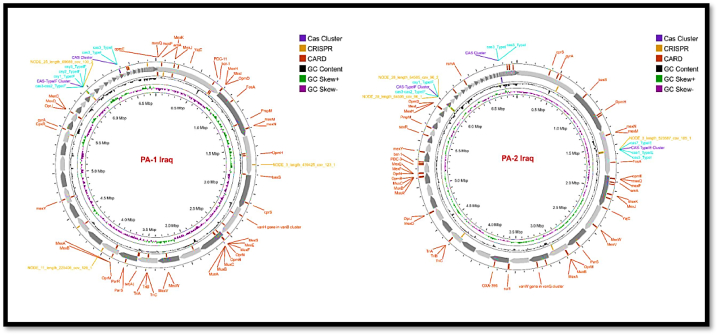
Circular genome mapping of clinical isolates of *P. aeruginosa* 1 (PA-1) and *P. aeruginosa* 2 (PA-2) showing resistant gene islands detected by CARD (red color); CRISPR in three loci (orange color); Cas system in three loci (purple color); phage in seven loci (sky blue color).

The G + C content of *P. aeruginosa* (PA-1) is 65.9 %, and the genome assembly size is 6,744,259 bp, divided into 106 contigs. There are approximately 6390 sequences in the genome that code for proteins, plus an additional 62 sequences that are non-coding RNAs (tRNAs and rRNAs included). At 50 % of the genome, the shortest sequence length is 268,940 bp, and the L50 count is 8, which is the least number of contigs whose length sum provides N50. On the other hand, the genome assembly size of *P. aeruginosa* (PA-2) is 6,921,241 bp and consists of a 65.9 % G + C content over 146 contigs. The genome contains 61 non-coding RNAs and 6707 sequences that code for proteins. The genome's N50 is 244,448 kb, and its L50 is 9. The circular genome representing the entire genome assembly is shown in Fig. 2.

**Fig. 3:**
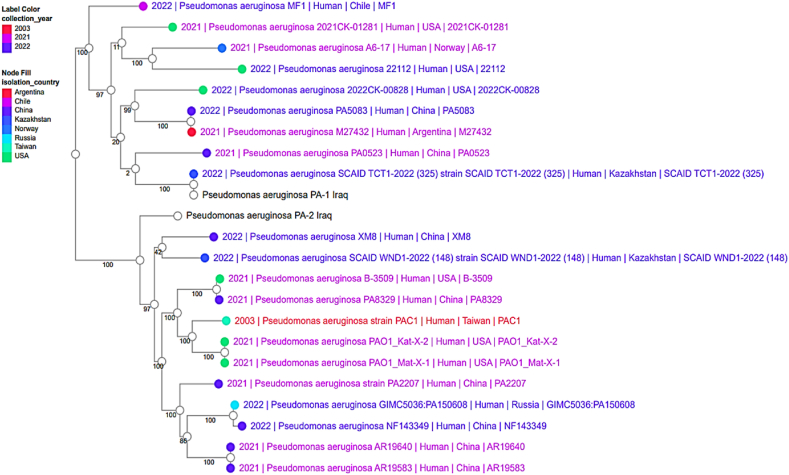
The phylogenetic tree, demonstrating the evolutionary relatedness of *P. aeruginosa* PA-1 and PA-2 to other strains of the same bacterium that have been subject to global selection. The tree was generated using PATRIC v3.6.2.

The distribution of the genome annotations is shown graphically in a circle (Fig. 3). This comprises the contigs, GC content and GC skew, CDS on the forward and reverse strands, RNA genes, CDS with similarity to known antimicrobial resistance genes, and so on, working from the outer to the inner rings. The subsystems to which these genes belong are indicated by the colors of the CDS on the forward and reverse strands.

#### 3.4.2 Antimicrobial resistance (AMR) profiling

Antimicrobial resistance patterns in PA-1 and PA-2 were characterized phenotypically. It was found that both strains have developed resistance to a significant number of antibiotics used in medical care. It is crucial to take into account certain AMR pathways, particularly either the existence or lack of SNP mutations that indicate resistance. Table 4 provides an overview of the AMR genes identified in the genomes, along with the relevant AMR mechanism.

**Table 4:** The distribution of sequence variants of antimicrobial resistant genes in both *P. aeruginosa* 1 (PA-1) and *P. aeruginosa* 2 (PA-2) against the eight groups of antimicrobial agents according to their mechanisms of action.

Antimicrobial resistance Mechanism	Antimicrobial resistant genes	
	PA1	PA2
Antibiotic inactivation enzyme	APH(3′)-II/APH(3′)-XV, CatB family, KPC family, OXA-10 family, OXA-50 family, PDC family	APH(3″)-I, APH(3′)-II/APH(3′)-XV, APH(6)-Ic/APH(6)-Id, CatB family, GES family, NDM family, OXA-50 family, PDC family
Antibiotic target in susceptible species	Alr, Ddl, dxr, EF-Tu, EF-G, folA, Dfr, folP, gyrA, gyrB, inhA, fabI, kasA, Iso-tRNA, MurA, rho, rpoB, rpoC, S10p, S12p	Alr, Ddl, dxr, EF-Tu, EF-G, folA, Dfr, folP, gyrA, gyrB, fabI, inhA, Iso-tRNA, kasA, MurA, rho, rpoB, rpoC, S10p, S12p
Antibiotic target replacement protein	FabG, fabV, HtdX	FabG, fabV, HtdX
Efflux pump conferring antibiotic resistance	EmrAB-TolC, EmrAB-OMF, MacA, MacB, MdtABC-TolC, MdtABC-OMF, MexAB-OprM, MexCD-OprJ, MexCD-OprJ system, MexEF-OprN, MexEF-OprN system, MexHI-OpmD, MexHI-OpmD system, MexJK-OprM/OpmH, MexPQ-OpmE, MexPQ-OpmE system, MexXY-OMP, MexVW-OprM, Tet(A), TolC/OpmH, TriABC-OpmH	EmrAB-TolC,EmrAB-OMF, MacA, MacB, MdtABC-TolC, MdtABC-OMF, MexAB-OprM, MexCD-OprJ, MexCD- MexPQ-OpmE, MexPQ-OpmE system, MexXY-OMP, MexVW-OprM, TolC/OpmH, TriABC-OpmH, OprJ system, MexEF-OprN, MexEF-OprN system, MexHI-OpmD, MexHI-OpmD system, MexJK-OprM/OpmH,
Gene conferring resistance via absence	gidB	gidB
Protein altering cell wall charge conferring antibiotic resistance	GdpD, PgsA	GdpD, PgsA
Protein modulating permeability to antibiotic	OccK8/OprE, OccK9/OpdG, OprB, OprB family, OprF, OccD1/OprD, OccD2/OpdC, OccD4/OpdT, OccD5/OpdI, OccD6/OprQ, OccD7/OpdB, OccD8/OpdJ, OccK1/OpdK, OccK10/OpdN, OccK11/OpdR, OccK2/OpdF, OccK3/OpdO, OccK4/OpdL, OccK5/OpdH, OccK6/OpdQ, OccK7/OpdD,	OprB, OprB family, OprF, OccD1/OprD, OccD2/OpdC, OccD3/OpdP, OccD4/OpdT, OccD5/OpdI, OccD6/OprQ, OccD7/OpdB, OccD8/OpdJ, OccK1/OpdK, OccK10/OpdN, OccK11/OpdR, OccK2/OpdF, OccK4/OpdL, OccK3/OpdO, OccK5/OpdH, OccK6/OpdQ, OccK7/OpdD, OccK8/OprE, OccK9/OpdG,
Regulator modulating expression of antibiotic resistance genes	OxyR	OxyR

#### 3.4.3 Phylogenetic analysis

The phylogenetic tree, which reveals PA-1 and PA-2's evolutionary relationships in comparison to other *P. aeruginosa* strains reported by the same or similar sources across continents in 2021–2023, is shown in Fig. 3.

As shown in this figure, PA-1 was clonally related to a strain from Kazakhstan, while PA-2 was unique compared to other strains. Both PA-1 and PA-2 are not clonally related to each other.

## 4 Discussion

The dissemination of antibiotic-resistant *P. aeruginosa* in hospital settings via multiple pathways is significantly contributing to the proliferation of resistance and of life-threatening infections within the community [20] The circumstances under consideration can be attributed, in part, to the indiscriminate and unregulated use of antibiotics to treat diseases. This practice exerts selective pressure on bacteria, hence facilitating the development of resistance [21].

In many bacteria, CRISPR/Cas systems provide adaptive immunity by targeting and cleaving foreign genetic elements, such as plasmids that carry antibiotic-resistant genes. In *P. aeruginosa*, an active CRISPR/Cas system could limit the acquisition of new resistance genes; however, if this system is present but inactive, the bacteria may still acquire resistance genes, leading to the emergence of multidrug-resistant strains. Reactivating CRISPR components could help limit the spread of resistance. Researchers may effectively kill or weaken resistant strains without the need to use broad-spectrum antibiotics by designing CRISPR-based antimicrobials that focus on resistant genes or essential virulence factors. For instance, delivering CRISPR-guided nucleases specifically to *P. aeruginosa* cell isolates could knock out resistant genes, restoring their susceptibility to antibiotics [22].

The research revealed a notable incidence of antibiotic resistance in *P. aeruginosa*, specifically in relation to numerous significant antimicrobial drugs [23]. The findings of the study indicated substantial resistance to β-lactam antibiotics, including cefazolin (87.0 %), ceftazidime (25.0 %), and ceftriaxone (18.0 %). This could potentially be due to the widespread utilization of these particular antibiotics by persons without adequate medical oversight [24]. It was discovered that the proportion of *P. aeruginosa* isolates exhibiting resistance to third-generation cephalosporins was 88.63 %. The prevalence of resistant isolates is consistent with other regional and international investigations. The findings presented in this study align with the observations made by Ref. [25], where it was shown that 88 % of organisms were resistant to ceftriaxone and 84 % to ceftazidime. In a study by Al-Kubaisy and colleagues (2020), there was an 88.2 % rate of ceftriaxone resistance and an 82.3 % rate of ceftazidime resistance among *P. aeruginosa* isolates. However, this study's results show that third-generation cephalosporin-resistant *P. aeruginosa* isolates are more common than was observed in Ref. [26], who found in northwest Pakistan that 54.35 % of isolates were resistant to ceftriaxone and ceftazidime.

The emergence of fluoroquinolones resistance in *P. aeruginosa* isolates can be attributed to the extensive utilization of these medications in recent times [27]. *P. aeruginosa* demonstrates the highest propensity for resistance development to multiple antibiotic classes, including quinolones, among gram-negative bacteria, according to the findings. This multidrug-resistant opportunistic bacterium poses a serious challenge to clinicians treating infectious diseases worldwide. Our study revealed that the highest rates of resistance were observed against cefazolin (88 %), and tigecycline (86 %), and that the prevalences of MDR and XDR isolates were 20 % (n = 20) and 11 % (n = 11), respectively. The findings of our study indicate that the prevalence of ciprofloxacin resistance in *P. aeruginosa* isolates was 43.0 %, whereas for levofloxacin resistance this was 42.0 %; this was not in agreement with El-Badawy and associates [28], who demonstrated distinct resistance ratios to levofloxacin (35.9 %) and ciprofloxacin (35.9 %).

The aim of the present study was to examine the resistance patterns of *P. aeruginosa* to various antibiotics. A total of 100 clinical isolates of *P. aeruginosa* were evaluated, each exhibiting distinct resistance statuses. PA-2 and PA-1 were characterized as extensively and pan-drug-resistant, respectively, following the finding through phenotypic analysis that both isolates exhibit resistance to numerous drugs, with the associated significant therapeutic implications; Table 3 reports the results of the genomic analysis of the resistance genes, which further substantiate the above. The frequency of blaPAO and blaOXA50, which are characterized as β-lactamase-resistant genes, has been brought to light in multiple publications in the literature on the characterization of the *P. aeruginosa* genome [23,29], and this does not make any difference in the *P. aeruginosa* strains under study. In our study, it is interesting to note that in PDR-PA-1 we detected the KPC-2 gene, which is usually only detected in *Klebsiella pneumonia*. This thus represents a unique and distinguished result in the genome of this strain in our country and the Arab nations; indeed, it is generally very rare, but has nevertheless recently been reported in other parts of the world [30]. On the other hand, in the genomic study on XDR-PA-2, a GES-type ESBL encoding gene has appeared, considering that it has not previously been reported in Iraq. Further, blaPAO, blaOXA-396, and NDM-1 carbapenemases are co-existent in this strain. This may be interpreted in terms of the unique resistance of this strain to all the (pan) antimicrobial agents used in our study. It was shown that blaOXA-50 may decrease the susceptibility of *P. aeruginosa* to ticarcillin, ampicillin, moxalactam, and meropenem. Thus, the presence of blaPAO, blaOXA-396, and NDM-1 carbapenemases, together within PDR- *P. aeruginosa* genomes, could explain its resistance to meropenem, colistin, and tigecycline [31].

The utilization of antibiotics has significantly transformed the approach to managing various infectious diseases. However, their escalating utilization, characterized by inappropriate and indiscriminate prescription practices, incorrect dosage administration and treatment duration, and the unrestricted distribution of antibiotics to the general population, has significantly contributed to the rise in antimicrobial resistance [32]. The notion of employing a synthetic CRISPR/Cas system as an antibiotic to eliminate specific bacterial genotypes was initially suggested several years ago [33]. Recent studies have demonstrated the efficacy of CRISPR/Cas in precisely targeting and removing drug-resistance-associated genes from bacterial populations, hence restoring sensitivity to antibiotics via the deletion of plasmids expressing AMR. However, prior to the application of CRISPR/Cas for targeting AMR in natural microbial populations, there remain various challenges that need to be resolved. To effectively harness the potential of this technology in mitigating the environmental and clinical dissemination of AMR through mobile genetic elements (MGEs), it is imperative to determine a feasible approach for such an implementation. This process's efficiency could be significantly enhanced through the direct reprogramming of CRISPR/Cas constructs to selectively target genes of particular interest. This kind of advancement could help fight resistance-building sites and possibly preserve or even boost the antimicrobial efficacy of current medications. By introducing a plasmid-containing CRISPR/Cas tailored to target a specific sequence unique to each genotype, individual bacterial strains can be removed from a population of mixed *Escherichia coli* genotypes, demonstrating the specificity of CRISPR/Cas antimicrobials. Two studies have shown that CRISPR/Cas9 may be delivered via phagemids, which are plasmids enclosed in phage capsids, to specifically kill two clinically significant bacterial pathogens: *Staphylococcus aureus* and *E. coli*. In one of these studies, phagemid transduction was used to deliver CRISPR/Cas9 constructs specifically designed to target AMR genes carried on plasmids, effectively eliminating these plasmids from the bacteria. Furthermore, the utilization of conjugative plasmids was employed as a means to facilitate the transfer of CRISPR/Cas9 to bacteria that harbor AMR genes within their chromosomes [34]. Previous studies have proven that this approach is efficient in re-sensitizing bacteria to antibiotics and in eliminating plasmids carrying AMR genes. It has also demonstrated sequence-specific CRISPR/Cas9 delivery to bacteria bearing virulence genes [35].

In the present work, a wide range of CRISPR/Cas system profiles were identified within our sample. The variability in the quantity, sequence, and size of CRISPR arrays can be attributed to their differential exposure to various bacteriophages across their lifecycles. This finding aligns with the research conducted by Jobayer and associates [36], who found that different bacterial species differ greatly in the quantity, length, and sequence of their CRISPR arrays [37]. The CRISPR/Cas systems play a role in restricting the introduction of exogenous DNA into bacterial cells and have also been associated with the modulation of virulence factor expression. Extensive research has been conducted on these systems across several taxa, encompassing both pathogenic and nonpathogenic species. However, the number of publications providing empirical evidence of such in terms of *in vivo* functionality remains limited [38]. This particular bacterium is considered one of the top five pathogens responsible for nosocomial infections globally and is classified as being within the ESKAPE group [39].

Hence, it is plausible to argue that the repertoire of spacers may serve as an indication or manifestation of bacterial lifestyle [40]. There has been surprisingly little research conducted in the Arab world, at least to the best of our knowledge, in this regard. The CRISPR/Cas system and its relationship to XDR-, MDR-, and PDR-*P. aeruginosa* were the focus of our investigation. Our study found that few or no antimicrobial agents effectively treat infections brought on by extremely resistant strains. Within the scope of this investigation, it was observed that a mere 6.0 % (6 out of 100) of *P. aeruginosa* isolates exhibited the presence of CRISPR/Cas, a proportion that is deemed quite low. Conversely, the prevalence of CRISPR1/Cas, CRISPR2/Cas, and CRISPR3/Cas among the isolates were determined to be 3.0 %, 6.0 %, and 0.0 %, respectively. The limited occurrence of the CRISPR/Cas systems seen in this investigation may be ascribed to the predominance of antibiotic resistance among the strains examined, resulting in their negative detection for these systems. A comparative analysis of our findings with those of other study laid down by Xu et al. [41], who revealed that, in Taiwan, the presence of the CRISPR/Cas system was detected in 30.7 % of clinical isolates of *P. aeruginosa*. By comparison, with those observed by Lin and co-workers [42], the CRISPR/Cas system does not seem to be widespread in *P. aeruginosa*, as evidenced by a recent study that indicated that just six out of 52 samples of the bacteria possessed it. Notable in this latter study is the way its findings corroborate previous research linking the CRISPR/Cas system to the absence of antibiotic-resistant genes. There appears to be a low incidence of CRISPR/Cas in *P. aeruginosa* as the system has been shown to be functional in isolates with full or draft genomes that are readily obtainable [43].

CRISPR/Cas was found in six out of 100 (6.0 %) clinical strains of *P. aeruginosa* taken from hospitals. The prevalence of the CRISPR/Cas system exhibits some considerable variability, with reported rates ranging from 30.7 % (54/176) to 12.4 % (27/217) [44]. A negative correlation was established between the presence of the CRISPR/Cas system and antibiotic resistance to certain antimicrobial agents. The findings of our investigation indicate that the isolates harboring this particular system exhibited reduced resistance to quinolones, aminoglycosides, β-lactams, and β-lactam/enzyme inhibitors. Richter and associates [45] found that a significant proportion of *P. aeruginosa* isolates harboring CRISPR/Cas exhibited susceptibility to a wide range of antibiotics; conversely, isolates lacking CRISPR were found to possess resistance to various medications [46] at which it could be deduced that the existence of this system is potentially linked to decreased drug resistance and, to a certain extent, may impede the acquisition of drug-resistance genes in *P. aeruginosa*. Liu, and co-workers [47] determined that carbapenem resistance in *P. aeruginosa* was strongly correlated with the presence of CRISPR/Cas loci. The identification of either CRISPR or CAS in antibiotic-resistant bacteria may suggest that these genetic systems were once present but subsequently lost in the course of the evolutionary processes to provide an opportunity for the bacteria to acquire AMR genes and develop resistance. Pinilla-Redondo and associates [48] observed that only a limited population of susceptible bacteria due to its cross-sectional design.

It is noteworthy that a significant proportion of the bacteria responsible for infections exhibit MDR, XDR, and PDR, which is a cause for great concern. Nevertheless, it may be argued that the absence of CRISPR/Cas in antibiotic-resistant bacteria provides support for our hypothesis that the presence of these systems reduces bacterial resistance to antibiotics. This suggests that the ongoing adaptation of bacteria and the occurrence of genetic recombination events are important to the bacterial response in the face of persistent antibiotic treatment. It underscores the necessity to conduct more extensive genomic investigations to analyze both domestic and global variants since CRISPR/Cas systems are not very common in antibiotic-resistant *P. aeruginosa* isolates. The study concludes that these systems can provide defense against the exogenous antibiotic resistance that can develop in the absence of CRISPR/Cas modules. In the future, these systems may be designed to act as weapons against germs that are resistant to antibiotics, which makes this development crucial. With the ability to precisely cleave bacterial genes and re-sensitize antibiotic-resistant cells, the CRISPR/Cas system offers fresh possibilities for eliminating MDR strains.

Phylogeny analysis of the phylogenetic relationship between PA-1and PA-2 with other strains revealed that the PA-1 under study has 100 % identity to those of human origin in Kazakhstan and it has some phylogenetic proximity to all other countries in the world, like Argentina, the US, Kazakhstan, China, and Norway. Long and associates [49] reported the genome sequence of a multidrug-resistant *P. aeruginosa* ST463 strain containing 23 ARGs in China. This clone has the potential to become a dominant endemic clone in eastern China. To prevent clonal dissemination, continuous surveillance is necessary in the future. On the other hand, Al-Tememe and co-workers concluded that the MLST is a typing used on several conserved housekeeping genes, namely acsA, aroE, guaA, mutL, nuoD, ppsA, and trpE, and the molecular confirmation by the aroE gene in medical microbiology is a rapid and cheap alternative to phenotypic methods of bacterial identification [50]. As a result of population dynamics, this may have occurred. Diseases and genetic material can be transferred from one community to another when populations relocate. Better economic mobility and social contact between the two areas explain the phylogenetic closeness of our isolates to the strains seen in Fig. 3 from the two countries. On the other hand, PA-2 was unique and different to all studied genomes of *P. aeruginosa* in the world over the last three years, allowing us to conclude that this strain is globally characteristic and very distinguished.

## 5 Conclusions

It was concluded that the CRISPR/Cas system and drug resistance are antagonistic to one another. Further, the whole genome sequencing for XDR- and PDR-*P. aeruginosa* revealed that they possess valuable tools (virulence, CRISPR, and resistant genes) that account for their ability to invade and persist in the host environment, suggesting that they represent a threat to public health and the seriousness of any associated illnesses. The challenges associated with managing such issues are significant. Furthermore, the variety of mobile genetic elements found in both strains reveals how active each strain was at different points in its evolutionary history. The whole genome sequences of *P. aeruginosa* PA-1 and PA-2 were better understood through the use of the WGS method. The presence of many resistant and distinct CRISPR/Cas system areas in these isolates' genomes raises concerns about their possible consequences regarding public health, the seriousness of the illnesses they could inflict on humankind, and the challenges in treatment that may arise. Differences in MGEs between the two strains show how active each was throughout its evolution.

## CRediT authorship contribution statement

**Hekmat A. Owaid:** Writing – original draft, Validation, Resources, Methodology, Data curation. **Mushtak T.S. Al-Ouqaili:** Writing – review & editing, Visualization, Supervision, Project administration, Investigation, Conceptualization.

## Ethics approval and consent to participate

The study participants provided verbal informed consent according to the questionnaire laid down by the medical committee of the ethics at the University of Anbar, Iraq, who gave their approval for this study. The university order, with approval number 14 of March 15, 2022, was used and carried out according to the Helsinki Declaration.

## Consent for publication

Not applicable**.**

## Availability of data and materials

Not applicable.

## Funding

None.

## Declaration of competing interest

The authors declare that they have no known competing financial interests or personal relationships that could have appeared to influence the work reported in this paper.

## List of abbreviations:

**CRISPR**: Clustered Regularly Interspaced Short Palindromic Repeats
**Cas**: CRISPR-associated protein
**MDR**: Multidrug resistance
**XDR**: Extensively drug resistance
**PDR**: Pan-drug resistance
**AMR**: Antimicrobial resistance
**PA**: *Pseudomonas aeruginosa*
**MGEs**: Mobile genetic elements
**MBL:**: Metallo-β-lactamase
**CR**: Carbapenemases
**PGFams**: PATRIC global protein families (PGFams)
**ESBLs**: Extended spectrum β-lactamases
**WGS**: Whole genome sequencing
**AST**: Antimicrobial susceptibility testing
